# Exploring nursing students’ reality shock and professional behavioral development in clinical practice: a hermeneutic phenomenological study

**DOI:** 10.3389/fmed.2025.1490975

**Published:** 2025-03-06

**Authors:** Feifei Cui, Yundan Jin, Rongting Wang, Jingya Zhang, Congying Jin, Fangju Xu, Songping Yang, Meiqi Yao, Shijian Han, Hangying He

**Affiliations:** ^1^Department of Nursing, The Affiliated Dongyang Hospital, Wenzhou Medical University, Dongyang, China; ^2^Department of Nursing, The Second Affiliated Hospital, Zhejiang University, School of Medicine, Hangzhou, China; ^3^Department of Quality Management, The Affiliated Dongyang Hospital, Wenzhou Medical University, Dongyang, China

**Keywords:** first clinical internship, nursing students, reality shock, stress perception, nursing behavior

## Abstract

**Background:**

The reality shock experienced by nursing students in clinical practice has attracted considerable attention. However, existing studies largely focus on quantitative analysis and overlook the depth of individual experiences.

**Objective:**

This study adopts a qualitative research method and utilizes hermeneutic phenomenology to examine in depth the reality of the shock faced by nursing students during clinical practice, to demonstrate its impact on the development of professional behavior and to provide empirical evidence to improve nursing education.

**Methods:**

A qualitative research method was adopted involving 30 nursing students completing a 32-week clinical internship at a tertiary hospital in Jinhua City. The subjects participated in focus group interviews conducted in three groups to collect information about their learning experiences during the internship. Wiklund’s hermeneutic-phenomenological approach was used to analyze and interpret the data.

**Findings:**

From the learning experiences of nursing students in clinical practice, three main themes of “promoting professional nursing behavior” were summarized: (1) Guidance from clinical instructors: The influence of preceptors’ words and actions on students’ professional behaviors; (2) Inspiration from clinical nurses: Observing experienced nurses enhances students’ professional identity; (3) Learning from real-life cases: Clinical cases help students understand the core values of nursing.

**Conclusion:**

Nursing students face emotional challenges and cognitive changes during clinical practice. Clinical instructors should instill positive energy in these students through professional behavior. They should encourage students to observe, analyze, and reflect on the exemplary words and actions of clinical nurses and to obtain authentic feedback through patient interactions to refine their professional behavior. This study can serve as a valuable reference for clinical teachers in supervising nursing students entering clinical practice for the first time and helping them develop their professional nursing behavior.

## 1 Introduction

School education is the primary route through which nursing students acquire basic theoretical knowledge and skills. However, the acquisition of theoretical knowledge represents only a fraction of nursing education, with a greater emphasis on the application of this knowledge in practical nursing contexts (1, 2). Consequently, clinical practices prove to be central nodes in connecting theoretical knowledge and practical nursing practice, enabling students to apply learned concepts in authentic clinical settings (3). Through these experiences, students encounter different pathologies and care scenarios, change their professional perceptions, promote appropriate professional behavior, and refine their clinical aptitude and decision-making skills (4, 5). The clinical experience serves as a time for students to build confidence, develop skills, and promote their professional development (6).

However, the clinical internship can present a variety of challenges and stresses, especially for aspiring nursing students. Pressures from patients, mentors, colleagues, and the clinical environment itself could impact learning outcomes and threaten psychological well-being (1, 3, 7–10). However, there remain few effective strategies to support and guide nursing students in coping with these realities of clinical practice. Therefore, a concerted effort toward deeper research and attention is warranted to continuously improve the quality of internship training and thereby improve the learning outcomes and professional development of nursing students (11).

## 2 Background

### 2.1 Challenges and coping strategies of nursing students

Reality shock refers to the intense emotional and psychological reaction one experiences when confronted with a reality that differs significantly from expectations (12). This phenomenon is often observed in individuals transitioning into a new environment or role, such as recent college graduates starting their careers or new employees adjusting to their workplace. Reality shock can lead to feelings of disappointment, confusion, and stress, which can even trigger anxiety or depression (13). Nevertheless, nursing students beginning their first clinical placement often express dissatisfaction with their clinical performance due to their lack of experiential foundation (14, 15). During the early stages of the internship, they face challenges and stress beyond those encountered in traditional classroom settings (12, 16). Research has shown that stress during an internship can impact nursing students’ physical and mental well-being (17). When stress levels in the housing environment become too high, it can cause anxiety responses that go beyond moderate levels (1, 3, 7, 12, 18), including feelings of helplessness, anxiety, and even manifestations such as insomnia and nightmares. This situation impaired their ability to learn and led to loss of self-esteem and self-confidence, which in turn impaired their chances of achieving clinical competence (6). The more negative the initial clinical placement experience, the more pronounced nursing students’ feelings of uncertainty and anxiety about their future placements, potentially impacting their learning outcomes and future career development (19, 20). Research suggests that chronic exposure to stress and failure to use effective coping skills can negatively impact their communication skills, thinking skills, interpersonal skills, and decision-making skills, and can affect their academic and clinical performance to the detriment of professional nursing behavior (8, 21). In particular, nursing students who demonstrate a positive attitude toward stress as a challenge can achieve growth-oriented benefits and increase motivation to learn (21, 22). Problems they encountered in clinical practice taught them to think about what they did, read, observed, and were taught, and enabled them to consider different perspectives rather than just a single point of view. Importantly, students were able to transfer the act of reflection to the clinical environment and thereby improve their own critical thinking behavior (23). Therefore, supervising faculty should prioritize nursing students’ personal growth and development during internship, including attitude toward the nursing profession, cultivation of professional behavior, and resilience in dealing with real-world challenges (5, 16).

### 2.2 Qualitative research: an in-depth study of nursing students’ internship experience

At present, most clinical internship studies at home and abroad are based on quantitative research methods, aiming to investigate the causes and levels of stress experienced by nursing students during internship and differences in internship stress among people from different backgrounds (1, 8, 12, 21). In contrast, few studies used qualitative research methods to understand nursing students’ subjective experiences during internship (20, 24, 25). However, as students’ personality traits develop, their low stress resistance, low frustration tolerance, weak sense of responsibility, and weak willpower become more and more apparent. Students completing a nursing internship for the first time must, in addition to mastering basic nursing knowledge and skills, also apply complex nursing theories and techniques to actual patient care. As a result, they face challenges such as dealing with intensive patient care, increased tasks and workloads, and increasingly complex professional skills. These challenges not only impact their learning outcomes, but can also affect their willingness to pursue the nursing profession in the future. Therefore, an in-depth exploration of students’ inner experiences and emotional aspects, cognition and professional experiences during this learning process is of great importance.

### 2.3 Social learning theory: designing nursing internship experiences

Social learning theory provides a robust theoretical framework for research in nursing education and enables a deeper understanding of nursing students’ internal experiences and coping strategies when faced with real-word challenges during an internship as well as their impact on their cognition, emotions and behavior (26, 27). According to social learning theory, individuals acquire new knowledge and behaviors through observation, imitation, and interaction with others. Nursing students can learn new behavioral patterns through observation and imitation and learn exemplary behaviors through social interaction, thereby increasing their self-efficacy (28). Self-efficacy, as postulated by Bandura (29), plays a crucial role in influencing the life course of individuals through their impact on the environment and the choices they make. Students who have a strong sense of self-efficacy tend to choose challenging endeavors that promote their educational growth and enhance their competencies, thereby equipping them with the necessary skills to excel in the nursing profession (30). Bandura (26) emphasizes that learning occurs through verbal guidance, sharing experiences, and observing the behavior of others in specific environments. This learning process reflects the interaction between behavior and control conditions. This theoretical perspective allows us to examine how the social environment of the nursing internship impacts nursing students’ experiences. For example, the interactions between nursing students and faculty, nursing staff, and peers during internship, as well as the power structures and social norms within the internship environment, can influence their behavior and attitudes.

## 3 Materials and methods

### 3.1 Design

This study employs a qualitative research design and utilizes focus group interviews and hermeneutic phenomenology for data collection and analysis. Healthcare professionals are increasingly using focus group interviews as a qualitative research technique, a trend that has been observed since the 2007 study by Halcomb et al. (31) and Rabiee (32). Focus groups allow for the inclusion of more participants, thereby promoting greater discussion and idea generation than individual interviews (33–35). Focus groups can provide insights into individuals’ thoughts and feelings on specific topics. These discussions effectively provide nuanced insights into individual life experiences and core beliefs and reveal different perspectives from different groups of people. They are unique in their ability to generate data based on the synergy of group interaction (32).

Hermeneutic phenomenology has both descriptive and interpretative elements (36, 37). It applies to uncovering a lesser-known or unknown phenomenon through an in-depth study of the lived experiences of those involved in the phenomenon (38–40). Phenomenology emphasizes the direct representation of conscious experiences. As a research method, it enables the return to and exploration of lived realities, while hermeneutics aims at the interpretation of texts and actions (41). This study aims to gain a deeper understanding of the inner experiences of nursing students, cognitive changes and their impact on the development of their professional behavior in clinical practice, hermeneutic phenomenology approach not only to describe the students’ actual experiences, but also to focus more on the participants’ perspectives and to explore in depth their subjective feelings and understanding of reality shock. For example, in-depth interviews with students at different stages of their placements were able to capture their subtle internal emotional changes and cognitive shifts. This paper is based on the Reporting Standards for Qualitative Research Reports (COREQ), see Supplementary Table 1.

### 3.2 Aim and research question

The aim of the study to uncover how nursing students’ behavior and attitudes are shaped by observation, imitation, and social interaction, and to analyze the effects of nursing students’ social context on these behaviors and attitudes.

How do observation, imitation, and social interaction during clinical internship shape the behavior and attitudes of nursing students, and what impact does the social context of these nursing students have on their professional development?

### 3.3 Setting and sampling

This study used a purposive sampling method to select nursing students who completed a 32-week clinical internship in a tertiary hospital in Jinhua City. The inclusion criteria were: (i) students who completed the clinical nursing internship with a passing grade, (ii) students who agreed to participate in focus group interviews, and (iii) students who were willing to sign a consent form. Data were collected from a total of 30 nursing students within 2 weeks of the end of the internship.

### 3.4 Research theoretical framework

**Figure 1:**
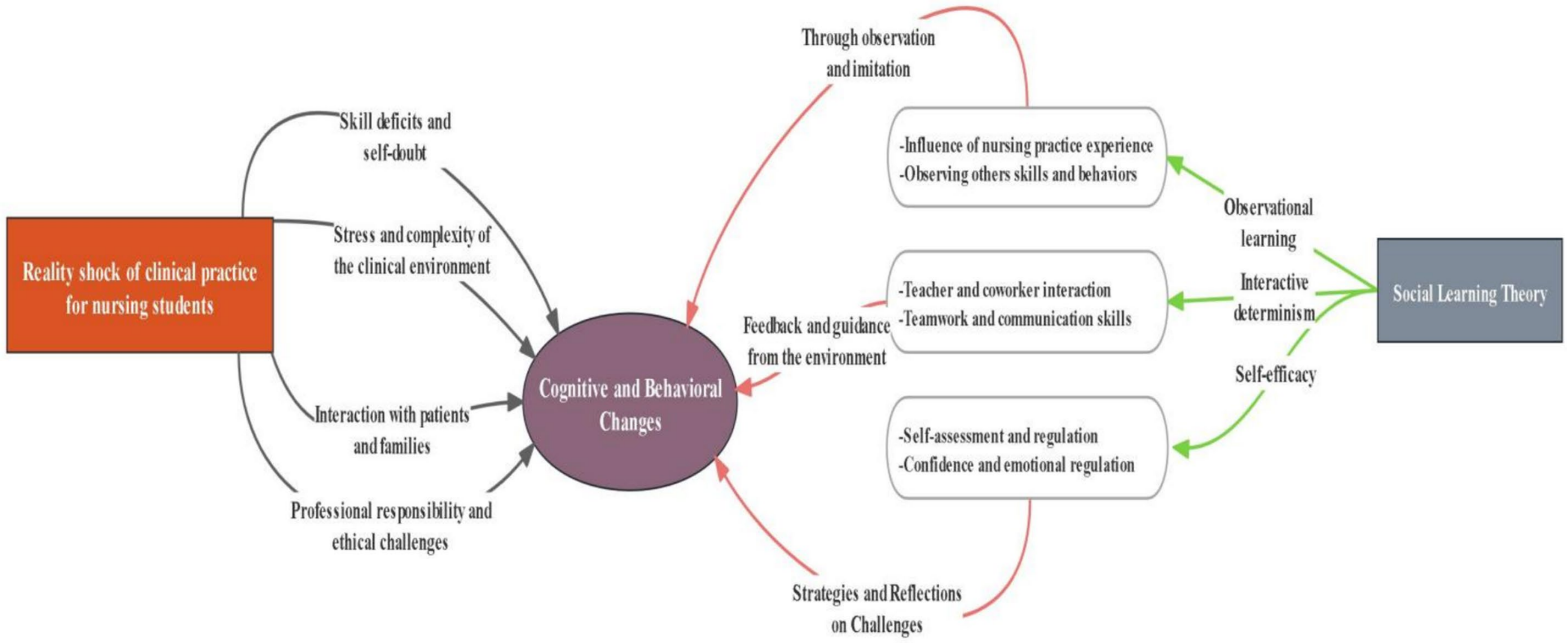
Diagram of the research theoretical framework.

This study adopts social learning theory as a guiding principle and influences the design of interview questions, data analysis and interpretation of results. Social learning theory includes four basic viewpoints: observational learning, triadic reciprocal determinism, self-regulation, and self-efficacy. This theoretical framework helps understand the impact of clinical practice on nursing students’ experiences, professional nursing behavior, confidence in professional development, and attitude formation. The research theory framework diagram can be found in Figure 1.

### 3.5 Data collection procedures

#### 3.5.1 Interview guide

The interview guide for this study was developed based on social learning theory, literature review, research objectives and clinical teaching experience. It focuses on nursing students’ experiences in clinical practice related to observational learning, interactive imitation, self-regulation, and self-efficacy (see Table 1). The guide consists of two parts: “Basic Information” and “Interview Overview.” (i) The suitability, relevance and coherence of the questions were checked by two teachers with over 20 years of clinical teaching experience; (ii) three experts with qualitative research backgrounds and extensive clinical teaching experience were invited to review and modify the interview draft; (iii) The revised guide was used to conduct interviews with three nursing students. Based on their feedback, further changes were made to the interview draft.

**Table 1:** Guidelines of the interview.

During your clinical practice, have you experienced any reality shocks? Could you please describe your most memorable experience in this regard? *[Observational learning]*
When facing reality shocks, who helped you reshape your cognition (yourself, teachers, parents) and ultimately enabled you to complete your internship tasks? *[self-regulation]*
Before and after your clinical practice, do you have any new perspectives on the ordinary and challenging aspects of nursing work? Do you feel a sense of accomplishment when patients are discharged after their recovery? *[Triadic interaction]*
What are your gains from experiencing reality shocks? Do you feel confident in working hard and becoming an excellent nurse after graduation? *[Self-efficacy]*

#### 3.5.2 Qualitative interview protocol

Upon confirming that the study participants met the inclusion criteria, the purpose and procedures of the research were communicated to the students both in writing and orally. Furthermore, it was assured that the interviews would be recorded solely for data analysis purposes and that the content of the audio recordings and field notes would remain strictly confidential and the information obtained would be used exclusively for this research. Interviews were conducted in a familiar and quiet discussion room, with students seated comfortably around a conference table to facilitate visibility and hearing of participants. The interview session lasted approximately 120 min.

To allay students’ concerns and elicit their genuine feelings, interviews were scheduled after students completed their clinical placements and exams. Three master’s students with a nursing background and qualitative research experience were selected as interviewers. Open-ended questions based on the interview draft were used to allow the nursing students to freely express their feelings and thoughts without being interrupted by the interviewers. If necessary, the interviewers provided appropriate information about the research topic. Nursing students’ freedom of expression was respected and ensured that each participant had ample opportunity to express their viewpoints. When nursing students’ responses were short, interview techniques such as “How did you feel at the time?” and “Could you elaborate on certain points?” were used to gather additional information. The researchers maintained neutrality and refrained from evaluating different perspectives in the interviews. Throughout the interview process, the interviewer’s assistant observed the respondents’ nonverbal behavior such as facial expressions, body movements, and tone of voice.

### 3.6 Data analysis

An interpretive phenomenological perspective was used to transform the raw interview transcripts into several meaningful clusters for similarity analysis and difference analysis to form a comprehensive narrative. Thematic analysis was used to interpret and synthesize the interview data. The themes of this study were identified using hermeneutic phenomenological data analysis techniques (42, 43). The analytical approach consisted of three main phases: surface interpretation, explanation and deep interpretation (42), for details see Table 2. Data analysis was carried out within a week of completing the interview data collection. The specific data analysis process is briefly described as follows: (i) The researcher read the interview data repeatedly to obtain a general overview of the content of the interviews and made notes on the page. (ii) Researchers independently analyzed the connections between their notes to identify and define themes. (iii) Researchers came together to compare their subjects. The unavailability of new themes was an important indicator in determining data saturation. (iv) Interpretive explanations of themes were recorded and quotes reflecting these explanations were selected (40).

**Table 2:** The hermeneutic phenomenological data analysis techniques.

Step	Hermeneutic method (43)
Surface interpretation	• Reading through a story to obtain spontaneous interpretations based on one’s own preunderstanding • Elaborating the interpretations by relating them to a theoretical framework • Contextualizing them in the interviewees’ life • Relating the results to the theoretical framework again
Explanation	• Validating the interpretations by structural analysis that reveals a plot whose implications are seen through the theoretical framework • Rewriting the story to accommodate the validated interpretation and presenting it for further validation by the interviewee • Identifying a metaphor that reflects the world of the text • Relating the metaphor to the interviewee’s life and the theoretical framework to gain deeper understanding • Challenging the result by re-reading the story and elaborating a new interpretation, if found, with the theoretical framework
Depth interpretation	• Merging all interpretations to open up a new perspective

### 3.7 Construction of the journey map

Journey mapping is a relatively new approach in medical research. Nursing student clinical practice journey maps are able to visualize the interaction of nursing students with various elements of the healthcare system, and can integrate the changes in emotions, behaviors and needs of students in practice, in-depth analysis their touch points, pain points, etc. To determine satisfaction points during practice, and to further derive opportunity points in order to optimize the experience. By constructing the framework of the journey map of nursing interns facing the impact of reality of clinical practice and adding the relevant data and information from the interviews, we have further clarified the growth path of nursing students under the influence of the reality of clinical practice and visualized it through the journey map.

### 3.8 Validity, reliability, and accuracy

To ensure the trustworthiness and validity of the study, the criteria suggested by Lincoln and Guba (44) were used, including credibility, confirmability, reliability, and transferability. Credibility: Within 48 h of the interviews, research team members transcribed the recordings verbatim. Two clinical nursing education experts with experience in qualitative research collaboratively reviewed the transcriptions to verify accuracy. Confirmability: The electronic transcripts were returned to participants for review, increasing the credibility of the research findings. Researchers and collaborators repeatedly reviewed the original data to ensure consistency and stability and developed results through induction and analysis. Transferability: Participants were interviewed in natural settings using open communication techniques so that research findings were applicable to similar research contexts.

### 3.9 Ethical issues

This study received approval from the Medical Ethics Committee (No: 2024-YX-252). To ensure the privacy and confidentiality of the students, the data was analyzed and presented anonymously. Participant data were anonymized by assigning unique numerical identifiers ranging from S01 to S30. In the participants’ invitation letter, nursing students were provided with comprehensive information about the research objectives, data processing procedures, confidentiality assurances, and anonymity guarantees. It was expressly stated that nursing students have the right to withdraw their consent at any time without this having any negative consequences for them. Prior to participating in the interviews, all nursing students were required to read and sign a consent form that further clarified their right to voluntarily withdraw their consent at any stage of the study (45).

## 4 Findings

### 4.1 General information of the study participants

The sociodemographic characteristics of the participants were systematically categorized and quantified using appropriate statistical measures, including frequency counts, percentage distributions and means, as shown in Table 3. Participants were nursing students who had completed 8 months of clinical practice, aged 19 to 23 years. The sample consisted of 28 women and 2 men.

**Table 3:** Sociodemographic characteristics.

Nursing students *n* = 30	
Age mean (SD)	21.13 (1.17)
Sex *n* (%)	
Female	28 (93.33)
Male	2 (6.67)
Educational background *n* (%)	
Bachelor’s degree	25 (83.33)
College degree	5 (16.67)
Family background	
Only children	13 (43.33)
Professional distribution *n* (%)^a^	
Laborers	3 (10.00)
Farmers	18 (60.00)
Businessmen	6 (20.00)
Administrators	3 (10.00)
Emotional expression *n* (%)^b^	
Never confide troubles to others	1 (3.33)
Only confide worries when consulted by friends	2 (6.67)
Tended to confide in only 1–2 people	18 (60.00)
Actively talk about their problems	9 (30.00)
Frequency of communication with the preceptors *n* (%)	
Often	19 (63.33)
rarely	11 (36.67)
Frequency of communication with the parents *n* (%)	
Often	21 (70.00)
rarely	9 (30.00)

a The term “professional distribution” is used to define the spectrum of professional occupations that parents of students practice in clinical practice.

b Emotional expression primarily refers to the coping strategies nursing students use when faced with clinical challenges during their clinical practice.

### 4.2 The growth trajectory of nursing students

**Figure 2:**
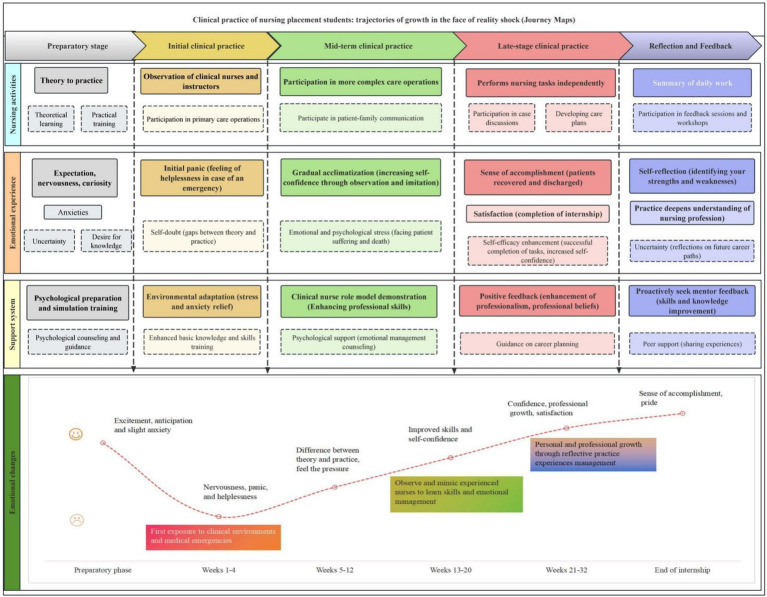
Clinical practice for nursing students: growth pathways under the influence of reality (Journey Map). A journey map typically consists of a horizontal axis (time axis) and a vertical axis (task axis). In this study, the horizontal axis of the nursing students’ clinical practice journey was divided into four phases: the preparation phase, the early clinical practice phase, the middle clinical practice phase, the late clinical practice phase and the reflection and feedback phase, in conjunction with the study group discussion. The vertical axis included nursing activities, emotional experiences and support systems used by nursing students during their practical journey according to the objectives of this study, and analyzed the key events in each phase according to the content of the interviews. Line graphs were used to represent visual trends in emotion scores over time. The horizontal axis represents time and the vertical axis represents emotional state, using a quantitative scoring system where a score of 1 represents a very negative emotion and a score of 10 represents a very positive emotion.

Based on the journey framework established in the desktop study, the interviews were studied in depth, and the storylines of different dimensions of nursing students going through different phases of their practice were drawn according to the different phases of nursing interns’ clinical practice were in view the care activities they carried out during practice, their emotional experiences and the support system that reconstituted the challenges of the multiple tasks they undertook, the emotional changes and the support they received at each stage of their journeys. A map of the growth trajectory of nursing students under the influence of reality was created, see Figure 2.

### 4.3 Qualitative results

#### 4.3.1 Overview

The analysis of the focus group data identified three main themes along with their respective sub-themes, as shown in Table 4.

**Table 4:** Sub-themes and themes obtained from interviews.

Sub-themes	Themes
-Overcoming initial panic -Emotional and Psychological support -Teach by example and Inspire potential -Shared insights and Mutual support -Feedback and Self-improvement -Adaptation and Reflection	Guidance from clinical instructors: The influence of preceptors’ words and actions on students’ professional behavior
-Silent Education -Realities of Nursing: Beyond Textbooks -Insights into Empathetic Nursing -Professional identity formation: challenges and growth	Inspiration from clinical nurses: Observing experienced nurses strengthens students’ professional identity
-Sense of accomplishment -Self-efficacy -Clinical growth journey	Learning from real cases: Clinical cases help students understand the core values of nursing

##### 4.3.1.1 Guidance from clinical instructors: the influence of preceptors’ words and actions on students’ professional behavior

Explanation: Clinical instructors provide nursing students with their expertise and behavior provide invaluable advice and support and help them to develop professional nursing behavior and attitudes.

###### 4.3.1.1.1 Overcoming initial panic

During their first encounter with emergency medical situations, nursing students often feel helpless and panicked, revealing a lack of psychological preparation. However, by observing the professional response and organized cooperation of the medical staff, they gradually overcome their fear, maintain composure and take on tasks within their capabilities.

“First time I encountered a patient who suddenly showed symptoms of respiratory distress, I became extremely panicked and helpless. Seeing the patient struggling to breathe filled me with fear and anxiety, and I was completely at a loss as to what to do. But the nurses and doctors around me were acted quickly and orderly…, I gradually began to calm down, comforted the patient’s family and helped organize medical equipment.” [A nursing student, S10].

Nursing students experience confusion and self-doubt in clinical scenarios. They recognize the gap between theoretical knowledge and practical application a lack of confidence in their abilities. This sense of experience is a challenge that many nursing students face and must overcome when transitioning into a real healthcare setting.

“I clearly remember feeling like an outsider at that moment, and feeling like I still did not know enough about healthcare work. I had acquired the relevant theoretical knowledge in class, I felt helpless and had no idea how to deal with a real emergency. I felt that my knowledge and skills were naive and inadequate to deal with such an emergency. This feeling of helplessness and self-doubt made me question my abilities and self-confidence.” [A nursing student, S03].

###### 4.3.1.1.2 Emotional and psychological support

Clinical instructors provide critical support to nursing practicum students in professional skills and emotional management to help them cope with the emotional challenges of nursing.

“They (instructors) not only guided me in my professional skills but also taught me how to manage my emotions and stress. The first time I faced a patient’s death, I felt very frustrated and helpless. My mentor patiently listened to me and gave me a lot of advice and psychological support to help me understand that this is an inevitable part of nursing and taught me how to recover quickly from such bad feelings.” [A nursing student, S16].

###### 4.3.1.1.3 Teach by example and inspire potential

Experienced clinical instructors have a significant impact on students by increasing their potential, confidence, professional skills, and career growth through effective knowledge transfer, teaching techniques, and supportive interactions. Good teacher-student relationships are crucial to the training of excellent nurses.

“My clinical instructor is a surgical nurse, he is very serious and careful and always gives me the opportunity to perform nursing procedures. He asked me for some expertise and then told me how the knowledge from the books could be flexibly applied to clinical situations…, he often showed me the details where it is easy to make mistakes and told me not to be afraid of making mistakes or doing a bad job, but to be brave enough to expose them so that I could learn a lot. When I make a mistake, he always smiles and makes me think first if there is a mistake in the operation, and then he shows me the correct treatment…, we understand each other very well, and when I encounter a difficult problem, I will take the initiative to consult him, and he always listens to me patiently and gives me advice and guidance. Overall, he is the friendliest teacher among my tutors, he gives me great psychological support and encouragement, which makes me feel comfortable.” [A nursing student, S15].

###### 4.3.1.1.4 Shared insights and mutual support

It is critical for nursing students to have the guidance of their clinical instructor and the support of their team. They can express their confusion and challenges in open discussion groups. By interacting and sharing experiences, nursing students not only solve problems, but also strengthen their teamwork skills and professional confidence.

“We will have a general clinical instructor who will bring us together every month (for shared decision-making and open-minded discussion) so that we can “spit out” the problems that arise in clinical practice. She will patiently analyze and solve common problems and guide us to encourage each other and share our experiences. In the seminars we often discuss and think together and help each other solve problems. Their presence makes me feel that I am not alone and that I can overcome difficulties together with them.” [A nursing student, S22].

###### 4.3.1.1.5 Feedback and self-improvement

Clinical instructors help nursing students progress in their practice by providing role modes, providing feedback, and asking encouraging questions. By teaching them to learn from their mistakes and emphasizing that mistakes are an opportunity for growth, nursing students can build self-confidence and promote their professional development.

“The most helpful thing was the direct demonstration and feedback from the clinical instructors. They observed while I performed surgery and after the surgery was completed, gave me specific feedback, telling me what I did well and where I could improve. They encouraged me to ask questions and take initiative to learn. There was a lack of expertise in some areas and the teachers suggested that I review the relevant knowledge I had learned in school and then answer these questions the next day. When I still did not understand them, they helped me with clinical scenarios. The advice they gave me was not to be afraid to make mistakes because every mistake is an opportunity to learn so that impact is more profound.” [A nursing student, S11].

###### 4.3.1.1.6 Adaptation and reflection

Despite hurdles such as fast-paced environments and emotional stress, nursing students demonstrate adaptability and resilience. They use self-regulation strategies and mentoring to turn challenges into opportunities for personal and professional development.

“I like to summarize. At the end of each day, I will review the day’s work and summarize what I did well and any problems. Then I will go through the textbooks to review the information to make up for the deficiencies, and I will consult with my clinical supervisor on issues that cannot be resolved with the book knowledge. I also set some short-term goals, such as improving specific skill, to maintain my motivation and concentration.” [A nursing student, S02].

“Although this experience brought some confusion and challenges for me, it was for also an opportunity for me to develop [determined look]. Shocks are inevitable in clinical practice, but by maintaining a learning attitude and taking positive actions, I was able to overcome the difficulties and continue to progress.” [A nursing student, S16].

##### 4.3.1.2 Inspiration from clinical nurses: observing experienced nurses strengthens students’ professional identity

Explanation: By observing and learning from experienced clinical nurses, students deepen their identification with the nursing profession and appreciate the meaning and beauty of nursing work.

###### 4.3.1.2.1 Silent education

Nursing students learn valuable things by observing the professional performance of senior nurses, you can expand your knowledge and skills and recognize the importance of remaining calm under stress and collaborating effectively. This silent training allows students to adapt and improve their behavior and performance.

“I experienced a tense and busy night shift In the emergency room as a flood of trauma patients streamed into the hospital following a road accident. There were patients with fractures, severe bleeding, pneumothorax, and cardiac arrest. The scene was full of screams and shouts… Many specialists arrived, and doctors and nurses rushed around, establishing intravenous access, performing various ancillary tests, and working tirelessly to care for the trauma patients. It was a very shocking scene, but I felt clueless and overlooked. My mentor was a senior nurse, who was knowledgeable in everything from cardiopulmonary resuscitation to trauma treatment and coordinated perfectly with the specialists. Her expertise and skills were very impressive and I admired her ability to remain calm under such pressure. Although I played no role in the rescue work, I watched and learned from their every move.” [A nursing student, S08].

“I worshipped their knowledge and clinical experience, and I would silently observe the teachers’ behavior to adjust and improve my behavior and performance (subtle influence, smile).”[A male nursing student, S01].

###### 4.3.1.2.2 Realities of nursing: beyond textbooks

On the front lines of healthcare, nurses demonstrate excellent communication skills, emergency management skills and teamwork, valuable lessons that books cannot convey in practical wisdom.

“I saw the reality of nursing and watched how they (nurses) communicated, interacted with patients and colleagues, deal with emergencies and worked as a team, all of which cannot be learned from a book.”[A nursing student, S14].

###### 4.3.1.2.3 Insights into compassionate care

In the hospital, the healthcare team treats patients with compassion and patience, instilling the power of respect and understanding.

“I was inspired by (their) compassion and patience with patients and their families: Compassion is not just about caring for the patient; it is about putting yourself in their shoes, listening to their stories and understanding their feelings. In this way, patients can feel respected and understood.” [A nursing student, S19].

###### 4.3.1.2.4 Professional identity formation: challenges and growth

The professional identity of nursing students in clinical practice is an important cornerstone of their professional development. When faced with clinical challenges, they gradually build their identification with the nursing profession through self-regulation and positive guidance from their clinical instructors. However, the process is not smooth; it is accompanied by a continuous exploration and reflection of self-esteem and professional roles. In practice, establishing this sense of identity often requires repeated review and adjustment.

“In the emergency room, long hours of standing, walking, and enduring repetitive tasks (taking vital signs, changing linens, etc.) made me feel like nursing was very hard work. However, through this experience I learned how to remain calm in a stressful environment. By observing how the teachers handled various emergencies quickly and efficiently, I gained a lot of experience and my skills in opening venous lines, cardiopulmonary resuscitation, and arterial blood gas collection improved significantly, while at the same time experiencing the importance of teamwork.” [A nursing student, S6].

Care goes beyond the mundane and requires professionalism and humanistic care. Nursing students deeply value the hard work and responsibility of nursing in practice.

“The mundanity of nursing work does not mean simplicity, and it requires a high level of professionalism and humanistic care from nurses… I helped a teacher care for a seriously ill patient who needed continuous monitoring. Turbulent emotions, details of medical care, and night work left me exhausted. The patience, care and skills required for nursing work far exceeded my initial imagination, and I became deeply aware of the hard work and responsibility of nursing, which also made me look at this profession more rationally.” [A nursing student, S30].

##### 4.3.1.3 Learning from real cases: clinical cases help students understand the core values of nursing

Explanation: Real-life clinical cases provide important learning opportunities for students and help them to learn the core values of nursing such as communication, teamwork, honesty, caring, empathy, and patient-centered care.

###### 4.3.1.3.1 Sense of accomplishment

The recovery of patients and the recognition of the medical staff are a deep validation of the efforts of the nursing students. This recognition serves as a powerful motivator, reinforcing their professional identity and pride, thereby reinforcing their ongoing commitment to their work.

“A middle-aged patient who was partially paralyzed due to a stroke was initially very frustrated and disappointed. With the careful care and rehabilitation training from medical staff, he gradually regained some of his functions and was eventually able to walk with the support of his family. I saw the patient go from despair to hope and finally recover and be discharged from the hospital. When the patient was discharged from the hospital, he specially wrote a letter of recommendation with the names of the instructor and me, … made me feel proud and satisfied.” [A nursing student, S25].

“By communicating with the patient and his family and the patient’s excessive care, I was able to give them a warm feeling during their hospital stay and received their recognition and gratitude when they were discharged from the hospital. This made me feel very satisfied and proud.”[A nursing student, S28].

###### 4.3.1.3.2 Self-efficacy

During their clinical practice, nursing students often encounter a spectrum of experiences that includes both positive and negative aspects. On the one hand, effectively completing complex tasks allows nursing students to adapt, realize their abilities, and increase their self-confidence. Conversely, resorting to avoidance or dependence when confronting challenges can promote self-doubt. These experiences highlight nursing students’ developing self-confidence and continued professional development.

“I feel like my potential and abilities go far beyond what I thought possible. I have learned to adapt my attitude and behavior when faced with difficulties and challenges… I encountered a very complicated nursing task and felt overwhelmed for a while. However, through hard work, study and communication with teachers and classmates, I finally managed to complete the task, which gave me more confidence in my abilities.” [A nursing student, S19].

“… I decided to run away or give up, when faced with a challenge instead of actively looking for ways to solve the problem. Once my teacher asked me to collect arterial blood gases, but I was not confident enough in this skill and was afraid that I would not be able to do it all at once; … I decided to ask other colleagues to help me with this task. The behavior of avoiding and relying on others made me doubt my abilities even more.” [A nursing student, S06].

###### 4.3.1.3.3 Clinical growth journey

Nursing students hone their technical and communication skills through immersive learning in various departments. Despite the challenges, their positive attitude and eagerness to learn advance clinical practice and promote continued growth.

“In internal medicine, I learned to mange the long-term care needs of patients with chronic illnesses; In surgery, I experienced the pre- and post-operative care process and perioperative management; In obstetrics and gynecology, I was involved in nursing care during labor and delivery and witnessed the birth of a new life; In pediatrics, I learned how to build good communication and trusting relationships with young patients and their families; and in the oncology department, I have deeply experienced the physical and mental challenges that cancer patients and their families face due to the disease. These practices have greatly benefited me and helped me improve my clinical nursing skills and professional behavioral qualities. Although I experienced some challenges and difficulties, such as stress, anxiety, frustration and self-doubt, I was also very fortunate to be able to learn and grow in clinical practice.” [A nursing student, S11].

## 5 Discussion

This study utilized a phenomenological approach to explore the reality shock experienced by nursing students during clinical practice. Qualitative analysis revealed students’ inner experiences and emotional responses when facing the gap between theory and practice, as well as complex clinical situations. For instance, students’ descriptions of their helplessness and panic during their first emergency situation provided insight into the nature of reality shock and its potential impact on their professional development. The findings indicated that the actions of clinical instructors, the role modeling of clinical nurses, and learning from real - life cases are crucial for shaping nursing students’ professional behaviors. These results suggest that nursing educators should provide positive guidance and support during clinical internships to help students cope with reality shock and promote professional growth. For instance, one student overcame fear of complex tasks and improved their skills with the encouragement of a clinical tutor, highlighting the effectiveness of positive guidance. These findings offer valuable references for clinical teachers to better understand students’ challenges and emotional fluctuations, enabling them to design more targeted teaching activities, provide psychological support, and enhance students’ adaptation to the clinical environment. For instance, teachers could organize group discussions and reflection activities to improve students’ teamwork and coping skills.

### 5.1 The influence of clinical instructors’ words and actions on students’ professional behavior: the integration of theory and practice

Clinical instructors’ words and actions had a profound influence on students’ professional behavior. Based on Bandura’s (26) social learning theory, this study found that clinical instructors’ behavioral demonstrations in clinical practice not only helped nursing students overcome initial panic but also promoted the establishment of their professional attitudes and the development of self-confidence. The expertise and behavioral standards of clinical instructors were internalized into their professional behavior by students through observation and imitation, thereby increasing their self-efficacy.

Positive feedback and guidance from clinical instructors were crucial in increasing students’ confidence and professional competence (5). However, the behavior of clinical instructors’ could also have negative effects. For example, negative behavior from clinical instructors could undermine students’ confidence and sense of professional identity (46) disrespect in the clinical learning environment affects students’ learning experience (47) anxiety-provoking teaching behavior in clinical situations is significantly associated with higher levels of anxiety and lower levels of mental health among nursing students (48). The following quote from a participant in our study exemplifies this phenomenon: “Without feedback, compliments, or praise from my instructor regarding my performance, I am uncertain whether my work meets the standards. Even criticism would be beneficial, as it would highlight my mistakes and enable me to correct them in the future.” This study reflected the suggestion that clinical instructors should respect students, avoid abusing them in public, and help them adjust their learning mindset through appropriate supervision and guidance to prevent anxiety or loss of confidence due to learning setbacks (49–51). Clinical instructors’ words and actions should be consistent with Maslow’s Hierarchy of Needs and meet students’ needs for respect and recognition. It is worth emphasizing the importance of sincere and constructive feedback, which not only helps correct students’ behavior but also motivates them to reinforce correct practices (52). This study further examined the role of clinical instructor behavioral demonstration in actual clinical scenarios. Compared to the “transformational model” proposed by Chikamori et al. (53), this study emphasized the importance of clinical instructors’ specific behavioral demonstrations in medical emergency situations on students’ psychological adjustment process. Kolb’s theory of experiential learning (54) was also validated in this study, emphasizing that nursing students internalized professional behavior through a process of concrete experience, abstract conceptualization, and active experimentation, facilitated by the recognition and approval of clinical instructors.

By integrating the above perspectives, this study provided in-depth insights into nursing education and highlighted the critical role of clinical instructors in shaping students’ professional behavior as well as the importance of applying theoretical guidance in clinical practice.

### 5.2 Observational learning and professional identity: the path to professional development for nursing students

By observing experienced clinical nurses at work, nursing students not only learned specific nursing skills and coping strategies, but also deepened their sense of belonging to the nursing profession and their professional identity. Observing experienced nurses helped students understand the complexity and diversity of nursing work. Through observation, students could learn to build trust with patients, manage emergencies, and communicate effectively as a team. This process of observational learning was a crucial way for students to understand and apply theoretical knowledge, helped them better adapt to their professional roles, and was crucial to the formation of their professional identities. The importance of observational learning was recognized by Arreciado Marañón and Isla Pera (23) and Bandura (26), who emphasized the importance of role models in practice in the construction of students’ professional identities as well as the process of individual learning and imitation of behaviors and their results through observation. However, unprofessional or negative behavior by clinical nurses could negatively impact nursing students, highlighting the need for hospitals to strengthen clinical nurse education (55).

One of the key findings of our study is the significant impact of clinical instructors on the professional development of nursing students. For example, a student described how his clinical instructor, a surgical nurse, not only provided technical guidance but also encouraged him to apply theoretical knowledge flexibly in clinical settings. The instructor’s supportive and patient approach helped the student build confidence and develop a strong professional identity. This study also found that nursing students continually refine and perfect their professional behavior through reflection and interaction with patients in actual nursing situations. By observing and imitating clinical nurses, nursing students, not only improved their professional skills but also improved their quality of life, gradually overcoming gender barriers and thus strengthening their professional identity. The study by Nasrin et al. (56) also supports this view and finds that nursing staff with role model qualities can stimulate nursing students’ motivation to learn. Emotional support was also essential to nursing students’ development, as clinical instructors’ empathy and support positively impacted students’ stress management and emotional coping (7). Social learning theory also emphasizes the role of model learning in providing pathways for skills, beliefs, attitudes, and behaviors (27).

The development of nursing students’ professional identity and behavior was a complex process involving multiple factors. Through observational learning, the influence of professional role models, adaptation to the clinical environment, and emotional support from clinical instructors, nursing students could continually grow in clinical practice and ultimately develop a solid professional identity and effective nursing behaviors.

### 5.3 Clinical case teaching: promoting the integrated development of nursing students’ professional skills and values

This study found that actual clinical cases not only provided students with the opportunity to apply theoretical knowledge in practice but also improved their clinical skills by working on real patient cases while promoting empathy and responsibility. Confronting ethical and emotional challenges in nursing practice enabled students to gain a deeper understanding of the core values of nursing, exceeding the impact of traditional theoretical learning.

Direct involvement in real cases significantly improved students’ practical skills and clinical decision-making ability (7, 57). The opportunity to interact directly with patients promoted the development of comprehensive skills. The importance of case-based teaching to help students deal with complex clinical situations (3). By engaging with real cases, students would be able to better understand patients’ needs and develop a spirit of humanistic caring, although this may involve some stress and anxiety. Proper guidance and support could help students overcome these challenges (58). The study also found that students established nursing ethics such as honesty, fairness and caring and demonstrated ethical judgment skills during actual clinical care, mirroring the content emphasized in school-based nursing ethics courses (59, 60). However, they also experienced moral distress in their clinical practice, which could lead to professional burnout if not handled properly (61). This finding is consistent with Jormsri et al. (62), who highlighted the importance of using appropriate strategies of honesty and deception based on specific situations in clinical care. Through clinical practice, students learned to embody different ethical views in nursing behavior, a point also reflected in LaSala (63) study, which found that case-based teaching helped students understand the core values of the nursing profession. For example, one student in our study described how her mentor provided emotional support following the death of a patient, helping her to understand that such experiences are an inevitable part of nursing. This emotional support is essential for students to develop resilience and cope with the emotional demands of the profession.

D’Souza et al. (64) emphasized the importance of diverse interactions in clinical nursing education and noted that feedback from clinical instructors, healthcare teams, or clinical patients played a key role in shaping nursing students’ professional behavior. These interactions were crucial for students to assess whether their behavior was consistent with job requirements in the workplace and to prompt them to continually adapt their behavior during the internship.

The clinical case teaching method provided nursing students with a comprehensive and in-depth learning platform while promoting the development of their professional skills and values. This method laid a solid foundation for them to become nurses with both technical competence and a spirit of humanistic nursing.

## 6 Limitations

Bandura’s social learning theory pointed out that the effectiveness of learning and behavior cultivation was influenced by the interaction between the environment, individual characteristics and behavior. The limitations of this study reflected the complexity and diversity inherent in this theory. First, various factors such as the internship environment of different hospitals, the teaching styles of clinical instructors, hospital management practices, and the individual characteristics of nursing students such as personality, learning styles, prior knowledge, and experiences could influence the outcomes of learning and behavioral development. However, only nursing students from a tertiary hospital in Jinhua City were selected in this study, which limited the representativeness of the sample. Consequently, the results may not comprehensively reflect the experiences of all nursing students, affecting the generalizability of the results. Second, this study relied primarily on interview data and did not include other data collection methods such as observations or surveys, which made it difficult to quantify the results and may have impacted the completeness and accuracy of the results. Finally, data collection depended on students’ self-reports, which could potentially be subject to recall bias.

Future research could expand the sample to include nursing students from more regions and backgrounds and examine the influence of various individual characteristics on learning outcomes to provide more targeted educational strategies. To obtain more comprehensive data, future studies should integrate multiple methods, including behavioral observation, surveys, interviews, and diaries, and combine quantitative and qualitative research to fully evaluate nursing students’ learning outcomes and psychological adjustment processes. In future studies, we aim to delve deeper into the impact of individual differences on students’ behavioral development and their strategies for coping with reality shock. For instance, we will examine how distinct emotional expression types influence students’ responses to clinical challenges. Additionally, we will explore the role of family background in shaping their learning experiences and professional identities.

## 7 Conclusion

Through qualitative analysis, this study revealed the reality shock experienced by nursing students in clinical practice and its impact on the development of professional behavior. Clinical mentoring played a critical role in helping nursing students cope with reality shocks, improve their professional behavior, and improve their self-efficacy. Based on Bandura’s theory of social learning, the clinical instructor supported nursing students to gradually adapt to the clinical environment through demonstration and guidance and facilitated learning through observation, imitation, and feedback. Despite some limitations, the results provided important practical guidance for nursing educators and highlighted the importance of training clinical instructor in future nursing education to support the professional development of nursing students, improve their skills in clinical practice, and enhance their professional qualities.

## Data Availability

The original contributions presented in the study are included in the article/Supplementary material, further inquiries can be directed to the corresponding author.
