# Supplementary material for: Exploring nursing students’ reality shock and professional behavioral development in clinical practice: a hermeneutic phenomenological study
Source: Front Med (Lausanne). 2025 Mar 6;12:1490975. doi: 10.3389/fmed.2025.1490975 (PMC11922931; doi:10.3389/fmed.2025.1490975)
Supplement: Supplementary file 1 [file Data_Sheet_1.pdf]

Supplementary Table 1 Consolidated criteria for reporting qualitative studies (COREQ): 32-item checklist

No	Item	Guide questions/description	
**Domain 1 Research team and reflexivity**			
Personal Characteristics			
1	Interviewer/ facilitator	Which author/s conducted the interview or focus group?	Fei fei Cui, Rongting Wang, Jing ya Zhang, Cong ying Jin, Fang ju Xu
2	Credentials	What were the researcher's credentials? *E.g. PhD, MD*	Master of Nursing (CFF, RTW, JYZ)
3	Occupation	What was their occupation at the time of the study?	Nurse clinician
4	Gender	Was the researcher male or female?	Male (FFC); female (RTW, JYZ, CYJ, FJX)
5	Experience and training	What experience or training did the researcher have?	-This study was conducted by Feifei Cui, who has a master's degree in nursing, and who has extensive academic background and professional experience in the field of trauma, such as pressure ulcers, and lower extremity ulcers. The researcher received systematic training in qualitative research methodology, including completing the strategies and considerations of nursing mixed methods or qualitative research methods, and received a certificate related to evidence-based trainer at Dongyang Hospital of Wenzhou Medical University.<br>-In the past five years, the researcher had participated in several qualitative research projects, such as coping with ulcer-inducing symptoms in rural patients with lower extremity venous ulcers inner experience; Stress overload, influencing factors, and psychological experiences of nurse

			managers and published papers. He has the ability to conduct in-depth interviews, observations, and research. He has skills in in-depth interviewing, observation, and textual analysis, and has extensive experience in data analysis and critical thinking.<br>-All members of this research team were actively involved in continuous professional development activities, regularly attend academic conferences, and read the latest research literature to keep their knowledge and skills up to date. In addition, they have a strong foundation in teaching nursing ethics, have assisted in the completion of a training program on the application of the “Four Themes Approach” to phased training in enhancing the ethical practice of new nurses, and have demonstrated sensitivity and responsibility to ethical issues in several research projects.<br>-Rongting Wang, Congying Jin, Jingya Zhang, and Fangju Xu were responsible for conducting the focus interviews and preliminary analysis of the data in this research project, and worked closely with other team members to ensure the smooth progress of the study.
Relationship with participants			
6	Relationship established	Was a relationship established prior to study commencement?	-Prior to the commencement of this study, preexisting relationships existed between the researcher and the nursing students.<br>-These relationships were mainly established through pre-service training, ethics teaching workshop pathways and were professional and educational relationships.
7	Participant knowledge of the interviewer	What did the participants know about the researcher? e.g. personal goals, reasons for doing the research	-In this study, nursing students were fully informed of the researcher's background information prior to the start of the study. The researcher clearly communicated the purpose of the study and the reasons for conducting this study to ensure that participants had a clear understanding of the study.<br>-Nursing students were informed of the neutral role of the researcher,

			aiming to explore their experiences and perspectives on the reality shock they face during clinical practice through the use of focus interviews, to reveal its impact on the development of professional behaviors, and to provide empirical evidence for the improvement of nursing teaching methods.<br>-The importance of informed consent from the nursing students was also emphasized and it was ensured that they understood the content and process of the study. Nursing students were also informed about how their data would be handled by the researcher and how their privacy and confidentiality would be protected.
8	Interviewer characteristics	What characteristics were reported about the interviewer /facilitator? e.g. Bias, assumptions, reasons and interests in the research topic	-The interviewers for this study were trained in qualitative research, had an in-depth knowledge of phenomenological methods, and had extensive practical experience in their teaching of ethics.<br>-The interviewers selected for this study were very interested in the research topic, had a deep passion for clinical teaching, and were committed to exploring new insights in the field. Their ability to demonstrate a high level of communication skills and trust building in their interactions with participants helped to create an environment of open and honest dialog.<br>-Interviewers during the study minimized the impact of personal bias through self-reflection and discussion with colleagues.
**Domain 2**<br>**Study design**			
Theoretical framework			
9	Methodological orientation and Theory	What methodological orientation was stated to underpin the study? *e.g. grounded theory, discourse analysis, ethnography, phenomenology, content analysis*	Phenomenology focuses specifically on the lived experiences of individuals and how these experiences constitute their understanding of the world. Phenomenology was chosen as the methodological basis for this study because we wished to explore the reality of the shocks faced by nursing students during their clinical practice, which involves deep insights into

			individuals' internal experiences.<br>Page 4, Line 132-154
Participant selection			
10	Sampling	How were participants selected? e.g. purposive, convenience, consecutive,snowball	-In this study, we used purposive sampling to select participants to ensure that the study would provide insight into the reality of the shocks faced by nursing students in clinical practice. We set clear inclusion criteria to ensure that participants were representative of the specific population we were studying. We aimed to recruit 30 participants, a number based on the expected data saturation of the focus interviews and the feasibility of student resources.<br>-During the recruitment process, we paid particular attention to the diversity of participants, including [age, gender, school, education, etc.], which helped us understand the research questions from different perspectives. We ensured that all participants provided informed consent before participating in the study and that their privacy and data security were protected throughout the study.<br>Page 5, Line 162-167
11	Method of approach	How were participants approached? e.g. face-to-face, telephone, mail, email	In this study, face-to-face interviews were conducted using a focus group approach.<br>Page 4, Line 131<br>Page 5, Line 191-193
12	Sample size	How many participants were in the study?	30 Participants<br>Page 5, Line 166-167
13	Non-partici pation	How many people refused to participate or dropped out? Reasons?	-During the recruitment process for this study, five nursing students refused to participate. The reason for refusal was the request of the school teacher to return to school early. All participated in the focus interviews during the conduct of the study and no one withdrew from the study.

			-In order to create a comfortable environment and encourage nursing students to speak actively, we provided fruits and snacks, drinking water, and prize incentives to ensure the flexibility of the interview time.<br>-Strict ethical guidelines were adhered to throughout the study to ensure that all participants were aware of their right to withdraw from the study at any time without suffering any adverse consequences.
Setting			
14	Setting of data collection	Where was the data collected? e.g. home, clinic, workplace	-The interview site for this study was chosen to be in the hospital's teaching conference room, which is large, beautiful, secluded, and an intimate and comfortable environment that helps to promote open and honest dialog.<br>Page 5, Line 191-193
15	Presence of non-participants	Was anyone else present besides the participants and researchers?	-During the data collection process of this study, in addition to the participants and the interviewer, there was also a recorder present as the interviewer's assistant, who was mainly responsible for doing only the work of recording the interviews, audio recording, observing and recording the participants' expressions, etc., and did not participate in the discussion work. Before the beginning of the study, the interviewer introduced the identity of the recorder.
16	Description of sample	What are the important characteristics of the sample? e.g. demographic data, date	-Thirty nursing students were selected to participate in interviews for this study based on inclusion and exclusion criteria. The sample size was determined based on the expected data saturation of the focus interviews and research resources. The sample included different ages, genders, schools, academic qualifications, etc. to ensure that the findings reflect diverse perspectives and experiences and help us to gain a deeper understanding of nursing students' experiences of facing real shocks in different contexts and the impact on professional behaviors.<br>-While our sample may not be fully representative of all relevant groups, it provides insights into specific subgroups.<br>-Demographic information was detailed in the table.

			Table 3<br>Page 7, Line 255-259
Data collection			
17	Interview guide	Were questions, prompts, guides provided by the authors? Was it pilot tested?	-In this study, the researcher crafted a detailed set of interview outlines for participants to explore the reality shocks nursing students face in clinical practice and the impact on patent nursing behaviors, with the aim of guiding discussion and ensuring consistency in data collection.<br>-To ensure the validity of these questions, the interview outline was critically reviewed and three students were selected for pretesting before the study began. The purpose of the pretest was to test comprehension of the questions, assess the feasibility of the data collection process, and collect feedback from the participants to revise the questions based on the feedback.<br>Page 5, Line 176-185
18	Repeat interviews	Were repeat interviews carried out? If yes, how many?	No
19	Audio/visual recording	Did the research use audio or visual recording to collect the data?	-In this study, we used audio recordings to collect data. This method allowed us to accurately capture what the participants were saying and the audio recording data was transcribed into text and used for data analysis along with field notes and other data to ensure accuracy. The transcription process was completed by trained transcriptionists.<br>-Before the recording began, all participants were clearly informed of the purpose of the recording and their informed consent was obtained. We emphasized our commitment to protecting their privacy and explained how the audio recording data would be securely stored and used.<br>Page 5, Line 188-190
20	Field notes	Were field notes made during and/or after	-The interviewer's assistant was responsible for taking notes in real time

		the interview or focus group?	during the focus group interviews in this study.<br>-Notes included, but were not limited to, body language, facial expressions, changes in tone of voice, and observations of participants' emotional states. These notes allowed for the capture of details that may have been missed when reviewing the recordings.<br>-Immediately after the interview, the interviewer recorded overall impressions of the interview and key points.<br>Page 5, Line 187-190
21	Duration	What was the duration of the interviews or focus group?	-This study utilized a focus group interview format, and we designed the interviews with data saturation in mind to ensure sufficient time to cover all relevant topics. An approximate duration of 120 minutes allowed us to explore participants' experiences in depth.<br>-Participants were informed of the expected duration of the discussion prior to the start of the interview and were provided with fruit, drinking water, incentives, etc. to increase their comfort during the interview.<br>-The interviewers managed their time effectively during the interviews to ensure that all key themes were fully discussed, while leaving enough time for in-depth exploration.<br>Page 5, Line 193
22	Data saturation	Was data saturation discussed?	Yes. Page 6, Line 217-218
23	Transcripts returned	Were transcripts returned to participants for comment and /or correction?	-The focus group members in this study established a WeChat group, where the interviewer compiled an electronic version of the transcribed text and returned it to the participants for them to comment and/or make corrections.<br>-In order to validate the accuracy of the data and to ensure that the data analysis was based on the most authentic and comprehensive participant experiences, participants were encouraged to suggest corrections to any inaccurate or incomplete sections, their feedback was taken into serious

			consideration, and the transcribed text was corrected accordingly.<br><br>-This process was clearly explained to participants before the study began and their informed consent was obtained.<br><br>Page 6, Line 236-239
**Domain 3 Analysis and findings**			
Data analysis			
24	Number of data coders	How many data coders coded the data?	-There were three coders involved in the data coding process in this study. The coders were trained on the research questions and coding techniques before they started coding.<br><br>-The coding process was conducted through independent coding, and when disagreements were encountered, they were encouraged to engage in open discussion and share ideas with the rest of the team in order to reach a consensus, aiming to improve the accuracy of the coding and to ensure that all relevant themes were fully considered.
25	Description of the coding tree	Did authors provide a description of the coding tree?	No
26	Derivation of themes	Were themes identified in advance or derived from the data?	-The theme identification process in this study was conducted based on Bandura's (1971) social learning theory framework.<br><br>-During the in-depth analysis of the interview data, a manual coding method was used to identify and generate emergent themes associated with social

			learning theory. These emergent themes were derived directly from participants' narratives and reflected their experiences and perspectives.<br>-The process of distilling new themes was conducted through a combination of participant feedback and team discussion.
27	Software	What software, if applicable, was used to manage the data?	Not applicable
28	Participant checking	Did participants provide feedback on the findings?	-The WeChat group was established at the beginning of the study, and the researcher provided an electronic copy of the transcribed text, a summary of the study's main findings and themes to the participants through the WeChat group format, allowing them 1 week to review and comment on the preliminary results to ensure that the findings accurately reflected the reality of what they experienced in their clinical practice.<br>-We emphasized the importance of their comments to improve the credibility and relevance of the study, and received some of the valuable feedback that helped to further validate and refine our conclusions.
Reporting			
29	Quotations presented	Were participant quotations presented to illustrate the themes / findings? Was each quotation identified? e.g. participant number	-In the results section of this study, we used direct quotes from participants to vividly illustrate our themes and key findings. These quotes provide a wealth of evidence for our analysis and help the reader gain a deeper understanding of the real-life experiences of nursing students during their clinical practice.<br>-We selected citations to demonstrate the diversity and depth in the data while ensuring that they accurately reflect the themes derived from the data.<br>-Each citation was identified through a unique participant number to protect participant privacy.
30	Data and findings consistent	Was there consistency between the data presented and the findings?	Data segments were carefully selected to reflect the theme of the study and were presented in detail in the findings section, which ensured a high degree of consistency between the data presentation and the findings of the study

31	Clarity of major themes	Were major themes clearly presented in the findings?	-The major themes have been clearly and unambiguously presented in the findings section, subheadings and clear paragraphs have been used to differentiate the different themes.<br>-Themes have been presented through detailed textual descriptions and diagrams to help readers understand better.<br>Table 4<br>See the results section of the article
32	Clarity of minor themes	Is there a description of diverse cases or discussion of minor themes?	In addition to the major themes, we also provided descriptions of secondary themes. The descriptions of the diversity cases showed how the research themes manifested themselves in different contexts and were able to provide a fuller picture of the participants' experiences. These secondary themes revealed other important patterns and insights in the data that help us understand the research questions more deeply.<br>Table 4<br>See the results section of the article
